# Federal Enactment of Healthy Homes Legislation in the United States to Improve Public Health

**DOI:** 10.3389/fpubh.2016.00048

**Published:** 2016-03-24

**Authors:** Alesia Coralie Ferguson, Christopher Yates

**Affiliations:** ^1^Environmental and Occupational Health, University of Arkansas for Medical Sciences, Little Rock, AR, USA; ^2^Green Bay Packaging, Arkansas Kraft Division, Morrilton, AR, USA

**Keywords:** healthy housing, home hazards, housing policy, asthma, home injuries and falls

## Abstract

Not all homes across America are “healthy” homes. This contributes to the poor health of Americans and exacerbates existing health conditions costing millions each year in health-care cost. Newer research is being conducted into strategies to alleviate biological, chemical, and physical hazards in the home, and various programs exist to assist the homeowner in making improvements in the quality of their home. Not every homeowner or renter nationwide or within community localities has access to these strategies or programs that could potentially improve their home environment and therefore the health of their family. The objective of this article is to propose elements of a policy to address this inconsistency and variation. This proposal centers around the federal enactment of a national policy demanding that each state implements a healthy homes program tailored to fit their specific state housing and health needs. Members of Congress from States that have successfully implemented healthy home programs should champion this policy. Organizations that recognize the impact of housing on health should support the development of a national healthy homes strategy. This article will discuss the need, outcomes, stakeholders, and minimum requirements of such a policy.

## Introduction and Background

Americans spend a substantial percent of their time indoors. Extreme weather conditions of hot summers and cold winters caused by global warming, and the American infatuation with the digital entertainment home results in more time spent indoors. For the elderly, sick, and very young, this time is spent in the residential home. Even for the working adult who previously spent 19% of their time working from the home in 2003, now spends 23% of their time working from home (1). With more time spent in homes engaged in leisure activities, our concerns sometimes surround a lack of activity and an increasing rate of obesity. However, other lurking health threats exist in the home. Many Americans live in “unhealthy homes” that may cause or contribute to multiple illnesses and injuries such as asthma, heart disease, broken bones, and cancer (2–4). In addition, there is socioeconomic disparity in the conditions of homes. In 2009, householders earning an annual salary of ≤$24,999 were almost five times more likely to live in inadequate housing than those earning ≥$75,000 (5). In the vein of a right to health insurance and basic human needs, guaranteed through programs, such as Welfare, Medicaid/Medicare, and the Affordable Care Act (ACA), every American should have the opportunity to have a healthy home, whether they rent or own. This article argues that it is time for Congress to ensure that every American understands the importance of a having a healthy home and pass legislation that requires every state to develop a statewide healthy home’s program. Each state program should promote and provide assistance to each resident in achieving a healthy home. Reasonable and basic standards should be the minimum mandated by the federal government to be the core of a state’s healthy homes program; however, the legislation should allow every state to tailor a healthy homes program to meet the specific needs within their state and to utilize existing state and local resources to the state’s best interest and ability. By effectively utilizing and coordinating existing State and Federal resources to reduce the incidence of adverse exposure and injuries in the home, this legislation can reap huge benefits in reducing health-care costs, improving comfort and satisfaction in the home, and maintaining home values.

An unhealthy home may result in adverse health outcomes such as respiratory illnesses (i.e., asthma), chemical poisoning morbidities and mortalities, infection, and bites (resulting in bacterial, viral, and other microbial disease transmission), injuries (i.e., falls and trips), depression, confusion, and discomfort. In terms of bacterial and viral transmission: (1) fleas transmit murine typhus and bubonic plague; (2) mosquitoes transmit malaria, dengue fever, chikungunya, and the West Nile virus; (3) ticks transmit tularemia, Lyme’s disease, and Rocky Mountain spotted fever; (4) house flies transmit dysentery, diarrhea, typhoid fever, and cholera; (5) cockroach transmit *Salmonella* and the poliomyelitis virus; and (6) rodents can transmit the hantavirus, Lassa fever, and salmonellosis to name a few (6, 7). Some of these are rare, common, or emerging diseases in the United States where the vectors (i.e., insects and rodents) need to be controlled in and around the home environment. Evidence from the Institute of Medicine suggests that cockroach and house dust mite allergens not only act as biological allergens for an asthmatic but also are causally related to the development of asthma (8). Chemical exposures that result from home building materials and residential use, overuse and improper use of cleaning, cosmetic, and treatment chemicals can exacerbate or promote a variety of illnesses (9). Property damage and loss of economic value directly impact the residents when the mold issue and pest issues (e.g., termite) result in permanent damage to the home structure. Indeed, some home problems stem from lack of resident initiative to fix and avoid problems (e.g., clutter affects the functionality of a home and can lead to falls, trips, fires, and pest infestation), but many homes problems also stem from resident lack of access to educational and functional local, state, and community resources.

The 2013 National Housing Survey discovered that 35% of homes have safety and housing hazards [i.e., have severe to moderate home deficiencies (10)]. These health and safety hazards range from ventilation, plumbing, and electrical problems to structural problems that influence indoor air quality and pest and mold problems. The 2013 Housing Survey fact sheet also illustrates that still close to 8% of homes have unsafe drinking water, over 9% have seen signs of rats in the last 12 months, and over 8% have leakage from the outside structure with the median year of home construction being 1974 (11). Homes built before 1978 could have lead-based paint, especially along window and door jams, and many have aging structures and appliances in disrepair.

The full impact and economic cost of substandard homes, and their resulting health-care and social costs in this country have not been thoroughly explored. However, data from some research illustrate huge potential savings and improved quality of life that can be realized by addressing housing issues in a targeted and comprehensive approach (12). Some common hazards stand out as primary targets. For example, still today, despite numerous legislations to curb exposure to lead hazards, over 450,000 children have blood levels above a safe threshold level of 5 μg/dl, where the percent of children lead poisoned in inner city and poor neighborhoods is higher (13). Directly targeting at risk communities living in old homes in need of repair can continue to lower those affected by lead poisoning. Asthma occurs in 8–10% of the population (more in poorer communities), where the annual cost for treatment attributable to dampness and mold in the home is estimated at 3.5 billion a year (14). Home intervention programs have shown success in reducing asthma outcomes [e.g., Ref. (15, 16)]. The occurrence of slips and falls that lead to fatalities, where the safety and design of the home environment plays a pivotal role (i.e., remove clutter and install handrails), grows as we age. In 2013, the direct medical costs of falls was 34 billion for the elderly over 65, resulting in lost family earning for mortality and substantial costs for disabilities (17). The National Center for Healthy Housing (NCHH) provides template of typical costs for addressing hazards in a home, demonstrating some hazards are potentially cost-effective (18). Still, the healthy homes research field in greatly in need of true cost-effectiveness evaluation to encourage and further guide the implementation of practical interventions in the home. In an economic analysis of the health impacts of housing intervention studies, Elisabeth et al. (19) found that of the 25 studies that reported some economic data, only one was considered a cost-effectiveness study and still lacked sufficient data for a complete analysis.

## Approach

The Housing and Community Development Act of 1992 (Public Law No. 102-550) was enacted to ensure that every Americans had access to a home to live in, at an affordable price (20). Since then, the Department of Housing and Urban Development (HUD) was assigned the task of providing assistance for rent or public housing for the poor (e.g., program such as HUD’s HOME funds). Some minimum standards have been established by HUD, Centers for Disease Prevention and Control (CDC), Consumer Products Safety Commission (CPSC), and the Environmental Protection Agency (EPA) in many areas of housing, including lead safety, weatherization standards, and even consumer safety on various products used in all homes. Research continues on the extensive dangers that can exist in a home, and now, within the last 10 years, there has been a growing realization of the impact home environments have on health, and in particular the influence of indoor air quality on adverse health outcomes, such as asthma and other respiratory problems. Specific steps have been taken to address healthy homes’ issues in this nation (Table 1), demonstrating the growing support for the problem. The home has come to be looked at in a *holistic fashion* and newer research on improved standards for building and maintaining a healthy home exist today. This should apply to homes rented or owned, or whether federal assistance is received for the home or not. The concern centers on the effectiveness of housing standards to result in change in the home. A consistent enforcement mechanism and wide dissemination are necessary for these standards to benefit the majority.

**Table 1:** **Specific efforts addressing healthy home**.

Year	Action	Outcome
1994	Presidential Executive Order 12898 for Environmental Justice addresses health and housing disparities among minority, low-income, and tribal populations	Highlighted risk factors (i.e., poverty) for lead poisoning in homes. Resulted in other federal agencies expanding their role to address other housing hazards
1999	HUD proposes a Healthy Homes Initiative (HHI)	Led to a preliminary plan to look at the key housing-related health and safety hazards in a home, and brought experts and practitioners together
2000	Collaboration from CDC and EPA to develop their own healthy homes programs	Led to a realization of each federal agency’s role in promoting healthy homes and a closer look at areas for collaboration
2004	National Center for Healthy Housing (NCHH) (some prior formation as far back as 1992)	Broaden and reinforced the collaborative effort between EPA, HUD, and CDC through a separate agency (NCHH)
2007	National Environmental Health Association offered Healthy Homes Specialist Certification	Highlighted the need for specialized training to address home hazards
2005	Healthy Housing Solutions, Inc. and the National Center for Healthy Housing (NCHH) operate the National Healthy Homes Training Center and Network through a cooperative agreement with CDC and support from HUD and EPA. The Training Center provides training through its network of partners around the country	Broaden the access to a variety of training courses for various professional across the nation (e.g., home inspector and nurses) and establishing local training centers in states
2009	Surgeon General Steven Galson issued “Call to Action to promote Healthy Homes,” including many actions for Government, home visitation programs, housing professionals, and other community organizations	Created greater awareness of the benefits of healthy home programs and the need for various professionals to strategically pool resources and address the most common home hazards of lead, mold, radon, and asthma triggers
2010	Renovation, repair, and painting rule (RRP) regulate contractor activities in target homes and child facilities. Other lead initiative to reduce childhood exposures occurred over the years	Led to the certification of 1000s of contractors nationwide and a greater awareness of lead-based paint hazards in homes. Has also led to a number of enforcement actions against contractors for non-compliance the rule. Other lead initiatives have potentially led to a reduction in lead hazards
2010	Merger of the Healthy Homes and Lead Poisoning Prevention Branch, within CDC	Led to the holistic approach to all hazards in the home and a means to grant federal funding *via* a comprehensive strategy. Stimulated state strategic plan developments as required by the grant requirements
2010	Centers for Disease Control and Prevention, National Center for Environmental Health, Division of Emergency and Environmental Health Services, Childhood Lead Poisoning Prevention Programs (CLPPPs) coordinate activities as they promote healthy homes programs	State strategic plans led to coordination among state and local agencies to form healthy home programs and develop referral and outreach program
2010	CDC Healthy People 2020 objectives align with health home concepts, 3 have to do with lead, 2 with radon, indoor allergen level (asthma), and monitoring diseases related to the home environment	These objectives will help guide federal and local programs, helping them focus on key areas, while enhancing surveillance of health outcomes
2013	HUD implements Federal Interagency Workgroup for Healthy Homes	Led to the development of a National Healthy Homes Standard. In the future, the standard will embody a Green and Healthy Homes Initiative. There is a now a growing recognition on the role of green materials and energy efficiency in a healthy home framework

*A number of initiatives have occurred over the last few decades to target efforts and promote interest in improving the quality of homes in America. Federal agencies, such as the HUD, EPA, and CDC, have led the way on many of these efforts (6, 21–24)*.

Implementation of a Federal Legislation mandating State healthy homes programs would further support healthy home efforts, where such a plan would force each and every State to address diseases, injuries, and unhealthy conditions related to substandard homes. Practical assistance exists to develop a State program. CDC offers a plan for building sustainable local healthy home programs and fostering partnership (22). Additionally, model programs also exists that target asthma reduction in the home and other disease outcomes [e.g., Ref. (25, 26)]. In addition, states, such as California, New Hampshire, Minnesota, and Connecticut, have been successful in implementing such state programs and have well developed and publically available strategic plans [e.g., Ref. (27)]. States that are lagging behind must learn from these proactive States.

Under this Federal Legislation, States should be given a 5-year period to enact a healthy homes program requiring a holistic and comprehensive consideration of all agencies, divisions, organizations, and bodies that have potential influence on the indoor home environment. This requires linking the already existing variety of programs throughout the State and coordinating efforts to assist residents and guide those who rent homes, retrofit homes, and inspect homes, in addition to training health agencies, visitation programs, and physician and care personal to recognize home-related illnesses (Table 2). A healthy homes program, therefore, also entails building the capacity for understanding healthy home aspects through recognized training methods/programs that focus on comprehensively addressing healthy home issues (12, 28). Such a comprehensive program is referred to as multi-hazard, multistep approach, where a variety of tools, resources, and referral processes for program managers, parents, and community members exists are utilized within the healthy homes program (3). Those states attempting to initiate or expand their healthy home programs are advised to look at all available state strategic plans and program experiences to formalize their approach and build on the program dimensions as presented in Table 2 [e.g., Ref. (27, 29, 30)]. In addition, HUD released it Health Homes Program Guidance Manual in 2012 with tools and successful case studies (31).

**Table 2:** **Dimensions of a healthy homes program**.

	Linking and evaluating existing program	Training and building capacity	Enforcing codes	Education and outreach
Who	Lead program, safety programs, asthma programs, gardening programs, poison prevention programs, drug buy-back program, federal housing programs, and tobacco programs	All groups, healthy homes specialist, contractors, landlords, physician, nurses, and realtor associations	State and local fire marshal, plumbing, electrical, and boiler inspectors	Residents (renters or owners)
What and how	Creating referral and computerized systems, conducting evaluations, and setting goals	Integrated pest management, lead testing and recognition, asthma intervention, and home safety and poisoning prevention, etc.	Zoning ordinances, housing, and building codes	Brochures, referral websites, workshops, school programs, and churches

*This table expresses some potential dimensions of a healthy homes program in addition to various stakeholders involved in and affected by the implementation of any State Healthy Home Program*.

For this policy initiative, there will be a number of stakeholders that will show interest or will be affected. The federal government should enact this state policy in partnership with state and local entities such as health departments (these are our policy suppliers). The State and its various agencies and organizations are expected to be the well-organized group with expert power. There are also various interests group who support healthy home initiatives, such as NCHH in part already supported through EPA, CDC, and HUD, and can provide support for trainings and strategies. Home owners, although demanders, tend to be less organized and have less political power to demand such a program. Outreach, education, and the provision of resources with this group will be required, and can have substantial benefits in this area to extend the sometimes limited reach in a person’s home. Behavioral changes, for example, can improve the indoor environment (e.g., proper use of stoves and individual units, clutter removal, and cleanliness) (32–34).

We expect some resistance to implementation of this policy from various professionals, including home inspectors, real estate agents, builders, and contractors related to expected increased costs and time. When looking at best practices and cost for making a home healthy, there is cost variation integrated into various levels of building, purchasing, renting, and retrofitting a home. In order to reduce persistent cost in the industry and in a healthy homes program, from the very moment a home is built, every consideration should be made to properly build the home to best practices (i.e., enforceable codes and recommended standards). The NCHH released its Standards for a Healthy Home in 2013, and it provides a comprehensive guide to all considerations for healthy home, including but not limited to moisture control, solid waste, pest management, electrical, chemical, and radiological agents, personal safety, and ventilation (35). Additionally, every time a home is retrofitted, every effort can be made to retrofit according to best practices, while utilizing any updated building and installation codes.

Today, the most recognized and comprehensive building codes are developed by the International Code Council (ICC), made up of a wide range of professionals and are free to be adopted by any locality (36). The International Residential Code (IRC) and the Building Code (IBC) applies to most building, plumbing, mechanical, fuel gas, and electrical requirements. Oftentimes, there is no penalty for contractors and landlords who violate any building code or provide less than substandard living, through a local or state entity, often as a result of lack of reporting, and in the case of the renter, weak state landlord habitability laws (37). Do-it-yourself homeowners are further left to their own devices. In general, contractors and residents do not fully understand the implication of their inappropriate actions in an indoor environment, and later costs to their health care or to the housing system.

Certainly, there is a competing proposal to the one proposed here, the *status quo*. This current approach relies on States to recognize the importance of home and health and voluntarily implement their own program or/and where the legislature passes minor policies that address specific hazards in a home (e.g., lead renovation and repair rules). History has shown this to not be fully effective and has resulted in a lack of coordination between programs, agencies, and bodies that influence the quality of homes and inconsistency in emphasis on the home environment across States. A federal approach is warranted, with the open recognition that barriers will need to be addressed systematically. Barriers to implementation of a state healthy homes program include but are not limited to (1) lack of coordination between affected agencies (state and local), (2) lack of coordination between medical entities and state and local entities, (3) lack of competencies to address healthy home issues, (4) lack of funding or ability to leverage existing resources, and (5) lack of focus and dedication from state legislature in the area of housing policy (38). Some of the barriers are driven by historical, social, political, and economic matters unique to each State’s environment.

In order for each State to be successful and address barriers to implementation, at a minimum, we suggest the Federal Legislation be written to provide States with needed support to conduct the following: (1) gather data on their home deficiencies in order to better target their programs; (2) provide initial training to state partners on how to implement program (help through CDC, EPA, and NCHH); (3) build capacity for healthy home inspectors and other needed professional, through training; and (4) develop a computer referral system that will save money in the long term and allow various entities to communicate and share data. There will be a need to continue to study potential barriers and understand special interests groups that might block passage. Wu et al. (39) provided an extensive analysis of other potential barriers and solutions (i.e., impediments and policy recommendation) to achieve healthy home environments that are applicable at a local level and should also be addressed (e.g., barriers: poverty, uneven distribution of benefits, and costs; solutions: broaden stakeholders and public recognition of achievements).

There should be a continual process of evaluation for each State program; its performance and its impact in reducing health hazards around the home and in improving population health should be addressed along with cost-effectiveness. Setting goals to reduce the level of hazards and injuries is suggested. Can a State program for example expect reasonably to see a 20% reduction in CO house poisonings and fires, a 10% reduction in asthma attacks attributable to the home environment, and a 25% reduction in fall and trips hazards around the home following a 5- to 10-year implementation of its program? Every State will decide what can be achieved given available State and Community resources, with some minimum expectation from the federal government.

## Conclusion

The key to having a healthy home is multidimensional and as defined by the NCHH requires adherence to seven principles (e.g., keep it … dry, clean, maintained, pest free, ventilated, safe, and contaminant free), with an eighth principle related to energy efficiency emerging (40). Adhering to these principles reduces exposures to multiple housing hazards that could make individuals sick. The enactment of a Healthy Homes Policy at the federal level will ensure that attention is given to an important issue, and a holistic program addressing resident, property owner, and home contractor behavior is developed along with avenues for state and local agency support. The Federal Legislation proposed here is a comprehensive approach to home health in a similar way the ACA addresses the many dimensions of health care, including provisions for Americans to be insured. In fact, the ACA now has provisions within Medicaid for coverage of lead follow-up and home-based asthma services, based on the effectiveness of such approaches. In mandating that each State sustain a healthy homes program and take actions to implement such a program, we are endorsing a harmonized and coordinated approach to the health of homes and families across this country (41). An appealing federal plan will develop the appropriate rhetoric and buy-in from important groups that would help lobby for such legislation. Potential members of Congress from the House Representative that would support, champion, and build momentum for Federal Enactment are likely to be those from States that already have successful healthy homes programs. Governmental strategies should be implemented in other countries and communities around the world, given geographical barriers and challenges are recognized.

## Author Contributions

AF: author responsible for concepts, manuscript design, and preparation. CY: author responsible for literature review, review of concepts, and manuscript editing.

## Conflict of Interest Statement

The authors declare that the research was conducted in the absence of any commercial or financial relationships that could be construed as a potential conflict of interest.
